# Unraveling the factors shaping academic success: A structural equation modeling approach for college students

**DOI:** 10.1016/j.heliyon.2024.e25775

**Published:** 2024-02-09

**Authors:** Wenwen Cao, S. Gnana Sanga Mithra, Aravind B R

**Affiliations:** a Department of Public Foreign Language Teaching, Qufu Normal University, No.80 Yantai Road, Rizhao City, Shandong Province, 276826, China; b Vinayaka Mission's Law School, Vinayaka Mission's Research Foundation (DU), Tamil Nadu, India; c Kalasalingam Academy of Research & Education, Krishnankoil, Tamil Nadu, India

**Keywords:** Self-efficiency, Stress, Academic performance, SEM

## Abstract

Academic success is a multifaceted achievement that depends on a myriad of factors, spanning personal, environmental, and institutional dimensions. The intricate interaction of numerous factors, such as how effective and interested a student is in their own academic performance, shapes their potential for academic achievement. This study's goal is to examine the effects that diversity, colour, and immigration status have on the academic accomplishment of 109 college students in Chinese province of Fujian. The main objective of the study to infer on how self-efficiency, self-interest, and stress affect academic achievement in particular. The researcher devised a survey tool in order to determine the degree of academic self-efficiency, academic self-interest, and stress connected to academic activities. The method of data collection that was used was called purposive sampling, and the participants were students in their primary year of university. The findings suggest that the scales that were used in the research have a high degree of reliability and exhibit very little inverse connection. “A Structural Equation Model (SEM) was created in order to examine the relative effects of stress and self-efficiency in predicting three aspects of academic performance: fresher man grade point average, credits earned, and persistence in studies beyond the first year (Considine and Zappala, 2002) [16]”. According to the data, self-efficiency is a stronger and more reliable predictor than the pressure connected with academic achievement. In conclusion, this study's originality lies in its holistic approach to understanding academic success, and its research implications extend to policy development, intervention strategies, equity and inclusion efforts, future research directions, and teacher training, all aimed at improving the academic success of diverse student populations.

## 1 Introduction

Academic success among college students is a complex and multifaceted concept, driven by an uncountable factors that extend beyond traditional notions of intelligence or knowledge acquisition. In the ever-evolving landscape of higher education, understanding what precisely contributes to academic achievement has become an increasingly pressing question. This study embarks on a comprehensive exploration to unravel the intricate web of influences that shape the academic success of college students. As students pursue their educational goals, they face a wide array of challenges, opportunities, and support structures that impact their journey. Some students excel academically, consistently achieving high grades and gaining a deep understanding of their chosen field. Others may struggle, facing barriers that hinder their ability to reach their full potential. The quest to understand the determinants of academic success transcends the classroom, extending into the broader socio-economic, cultural, and institutional contexts that envelop the college experience. This study recognizes that academic success is not solely a product of individual intelligence or effort, but rather the result of a dynamic interplay of various factors like self-efficiency, self-interest, and stress affect academic achievement in particular. The significance of this research is not limited to academic curiosity; it has far-reaching implications for policymakers, educators, and institutions seeking to enhance the academic experiences and outcomes of their students. By unpacking the various elements that influence academic success, this study aims to shed light on how colleges and universities can tailor their approaches to better support diverse student populations.

“Several researchers in the field of education have developed theoretical models to explain the relationships that exist between various aspects of learning and the educational results of students [[1], [2], [3], [4]].” These theoretical models take into consideration the qualities of the students, the settings in which they learn, and the quality of the education they get [5]. The results of an analysis of empirical studies on academic accomplishment and the factors that predict it suggest that the characteristics of the student have the most important direct influence on the outcome [6]. “Walberg's theory of educational productivity is one hypothesis of academic accomplishment that has been widely researched. This theory proposes that students' immediate psychological conditions and traits impact their cognitive, behavioral, and attitudinal educational results [7].”

“According to Walberg's research, there are nine crucial factors that influence learning outcomes, including student aptitude and prior success, motivation, age and developmental stage, quantity and quality of instruction, classroom environment, home environment, peer group, and exposure to media outside of the classroom [8].” “These elements include student aptitude and prior success, drive, age and developmental stage, quantity and caliber of instruction, and exposure to mass media outside of the classroom [8].” “Recent research on learning environments has mostly focused on the conceptualization and development of learning theories.” In contexts of higher education, student input is often included in assessments of both instructors and courses [[9], [10], [11]]. “Numerous studies have demonstrated the additive value of the psychosocial aspects of classroom learning environments in predicting student growth.” “These psychological traits, such as a student's overall performance, self-concept, attitudes, behaviors, and intrinsic motivation, are crucial for the evaluation of the curriculum and may provide details that might assist teachers in creating more effective learning environments. Constructivist-based measures of personal learning environments have been created and validated by academics [12,13]. These measures were intended to capture the unique perspectives that students bring to the classroom experience.

Numerous academics have conducted an extensive study of the factors that affect students' achievement at various educational levels. Take, for instance, the correlation between academic achievement, gender, and the overall performance of pupils in the UK [14]. “The economic and social standing of a student's parents has been shown to have an impact on the student's academic performance [15].” The economic and social status of the parents has an effect on the scholastic success of their offspring [16]. Additionally, there is a correlation between the socioeconomic conditions of one's parents (such as their income, level of educational attainment, and level of professional performance) and one's level of academic success [17]. The accomplishments of students in the past were connected to their success in the future [18].

“Students' academic success, satisfaction, physical and mental health, motivation, learning strategies, cognitive resources, and self-directed learning have all been linked to the quality of teacher-student interactions, classroom instruction, concentration, information processing, storage, retrieval, and learning [16].”

### 1.1 The impact of stress on academic achievement

The term "emotion" refers to a subjective state that is distinguishable by a person's physiologic emotions and reactions to particular circumstances, acts, and occurrences. “Academic feelings are intricately connected to a student's accomplishments, activities, and results [19].” In the beginning, academic emotions were broken down into three different categories: activating emotions (such as pleasure, pride, and rage), deactivating emotions (such as shame), and positive emotions (including enjoyment, pride, and hope) within the framework of the educational setting [20]. “Research has shown that having healthy emotions such as delight has a positive effect on one's academic performance [19].” On the other hand, undesirable deactivating feelings like fatigue may reduce motivation and interrupt information processing, which exemplifies the negative effect that such emotions have on academic accomplishment. “There is a considerable association between the good feelings that students experience and their performance, which indicates that pleasant emotions such as satisfaction, optimism, and pride are predictors of academic achievement [21].” Students' ability to do well on their midterm examinations is significantly correlated with their experience of positive activating emotions such as pride, joy, and hope [22]. Positive feelings such as happiness, optimism, and pride have been shown over and over again to be strong indicators of academic achievement in a variety of contexts, including academic performance.

### 1.2 The impact of self-efficiency on academic achievement

The degree to which one believes they are capable academically is an important component of academic accomplishment. It refers to the attitudes and ideas that students have towards their potential for academic achievement, and it also includes their confidence in their abilities to perform academic assignments and absorb the subject matter in an efficient manner.

Beliefs in one's own ability to succeed, or self-efficacy, play a part in the motivational process by encouraging people to put up their best effort, which in turn increases their level of commitment, persistence, and tenacity. “Students who have low levels of self-efficacy are more likely to believe that their failures are due to a lack of ability, while students who have high levels of self-efficacy are more likely to believe that their failures are due to a lack of adequate effort [19].” As a result, self-efficacy has an effect not only on task selection but also on task perseverance. Students who have a poor sense of their own capabilities are more inclined to avoid, put off starting, and eventually give up on their schoolwork owing to anxiety. On the other hand, there is some evidence from studies to indicate that metacognitive learning methods might be a factor in the association amongst self-efficacy and academic accomplishment. “More specifically, when confronted with obstacles, students who have higher levels of self-efficacy exhibit more tenacity and effort than their peers [16].”

“Despite the fact that self-efficacy has a positive effect on the quantity of effort put forth, there is evidence to suggest that students with greater levels of self-efficacy put forth higher-quality efforts than students with lower levels [30].” These students use deeper cognitive and metacognitive processing strategies, which enhance learning and academic success. “As a result, they have a high sense of self-efficacy. Students who have low levels of self-efficacy, on the other hand, have a tendency to prevent failure by selecting assignments that are simpler for them and prioritising surface-level strategies over deep learning techniques [15].

### 1.3 Correlation between stress and self-efficiency

The notions of stress and self-efficacy are intricately intertwined with one another. Self-efficacy is said to play a significant role in determining how one responds to environmental pressures, in accordance with Lazarus' cognitive model of stress [23]. Researchers have shown that people who have strong ideas of their own ability to succeed are inclined to see the expectations placed on them by others as barriers rather than as threats [[23], [24], [25]]. To put it another way, the degree to which an activity is viewed as stressful or scary is directly proportional to the individual's level of self-assurance in their capacity to deal with the circumstances. Therefore, self-efficacy regulates both the perception of extraneous pressures and the link between outside influences and psychological strain [26]. Self-efficacy also modifies the interaction between these two types of stress.

“According to the findings of the research, the appraisal of demands as either threats or challenges fully moderated the effect of academic self-efficacy on stress [24].” On the contrary, physiological levels of arousal that are connected with stress and anxiety give information that might alter an individual's appraisal of their own effectiveness [27,28]. In a similar vein, students' evaluations of their own effectiveness might be negatively impacted by stress and concern [29].

“These results suggest that thoughts about one's own self-efficacy and one's degree of stress are connected. Individuals who have strong beliefs in their own self-efficacy are better able to see difficult circumstances as manageable challenges, which in turn lowers their levels of stress [29].” On the other hand, excessive stress levels may have a detrimental influence on a person's perception of their own effectiveness. Therefore, it is essential to have a grasp of the link between stress and self-efficacy in order to appreciate how people perceive and react to the expectations placed on them by the surrounding environment.

The relationships between these factors are intricate. For instance, high levels of academic stress may lead to negative academic emotions, such as anxiety or frustration. Conversely, positive academic emotions may boost self-efficacy and contribute to better academic performance. Moreover, self-efficacy can serve as a buffer against the negative effects of stress, as students with strong self-efficacy may approach challenges with confidence and resilience. While existing research has explored the individual contributions of stress, academic emotion, and self-efficacy to academic success, there is a need for more comprehensive studies that investigate how these factors interact. Understanding the nuanced ways in which stress and emotions impact self-efficacy, and in turn, academic success, can guide the development of interventions and support systems aimed at improving students' overall well-being and performance.

### 1.4 Conceptual framework

A detailed framework for examining the influence that students' feelings have on their academic success may be found in the control-value theory of accomplishment emotions. This idea was developed by Pekrun and proposes that happy emotions have a moderating effect on cognitive, metacognitive, and self-regulatory behaviours, which in turn have an indirect impact on academic accomplishment. Emotions have the potential to influence academic performance through two basic channels: cognitive and motivational. Each of these channels is comprised of four different processes.

Within the cognitive route, an individual's performance may be influenced by their emotional state through three distinct methods. To begin, one's feelings may have an effect on their ability to remember and retain knowledge, regardless of how they are feeling at the time. This is referred to as mood-independent memory. Second, students' emotions may alter their cognitive and metacognitive learning techniques, which in turn affect how they approach and make sense of the material they are given. In the third place, our mental processes, such as attention, perception, and the processing of information, may be influenced by our emotional state.

On the other side, experiencing pleasant emotions is the outcome of applying learning techniques that are multifaceted, adaptable, and complicated, as well as engaging in self-regulation. Students often adopt more in-depth learning tactics and exhibit greater involvement in the process of learning when they are exposed to positive emotions, such as happiness and excitement. Students who actively participate in stronger educational practises tend to have higher academic accomplishments, which is contributed to by the characteristics listed above.

The control-value theory of accomplishment emotions, on the whole, sheds light on the complex link that exists between one's feelings and their level of academic success. Students need to be motivated to use effective learning techniques and to participate in self-regulated learning, and positive emotions not only have an influence on the cognitive processes themselves but also play a significant role in this process. Students have the ability to enhance their academic performance and learning experiences by developing knowledge of the power of emotions that are beneficial and developing strategies to harness that potential.

### 1.5 Hypothesis

H1 Academic performance is directly impacted by academic self-efficiency.

H2 Stress that is helpful to learning has an immediate impact on performance.

H3 The connotation between educational self-efficacy and performance is mediated by positive academic emotions.

### 1.6 Participants

Our research included a total of 109 college students from illustrious educational institutions located in metropolitan areas throughout the province of Fujian in China. These students were selected without regard to their immigration status, racial or ethnic origins, or skin colour. Undergraduates who mobile to school and frequently enrol in freelance classes make up a substantial component of this student body. These students are also more likely to be non-traditional, minority, or immigrant students. Only 25 percent of students at this school are able to complete their studies and get a bachelor's gradation within six years of enrolling, which is a serious cause for concern over the school's attrition rates. In order to address this problem, the primary emphasis of our research is on first-year students, who are often seen as being at the highest risk of falling out of college in their primary academic year.

The participants' ages, on the whole, did not substantially differ from those of the general population (p = 0.13), with the average participant being 20.7 years old. In addition, there are about the same number of women (0.73 percent) in the sample as there are in the general population (0.65 percent). In addition, the percentages of persons who identify as white, black, or Asian in the sample are proportional to the percentages of individuals who belong to these categories in the overall population.

### 1.7 Data

The students responded to a questionnaire that was split into two parts, each of which asked them about a different facet of their academic careers. Respondents remained required to answer queries about their demographics in the first section of the poll. Their age, gender, high school GPA, race or ethnicity, the primary language, their country, and their age at immigration for those who were born abroad were among these factors. In addition, respondents provided us with either their college ID numbers, which we utilised to request respondents' academic records after a year.

The second part of the survey included questions on academic pressure and how confident respondents felt in their own abilities. We came up with an original method in order to investigate in more depth the connection between these ideas and the influence they have on the results of academic endeavours. we developed a tailored research instrument to delve deeper into these connections. We began by reviewing the literature and creating a conceptual framework. We generated questions that reflected these concepts and received feedback from experts, refining the questions accordingly. Once our instrument was set, we obtained ethical approval for data collection and proceeded to gather information from a diverse group of college students. The collected data was then analyzed to uncover the intricate relationships between these factors, providing valuable insights for improving students' academic achievements and well-being.

The self-efficacy and stress levels of college-related activities were explicitly tested with the use of this tool. A portion of the tasks that were included in the instrument were chosen at random from previously recognised assessments of academic self-efficacy, such as the College Self-Efficacy Inventory or the Academic Milestones Scale [30]. In addition, students who attended the same institution but were not a part of our research offered comments on 18 additional activities that they described as stressful.

The survey included a total of 27 questions, and some of the things it asked about were "writing term papers," "raising questions in class," and "balancing school and work." On a Likert scale of 11 points, with 0 being the least stressful and 10 representing the most stressful, the participants were asked to assess the difficulty of each activity. Respondents rated their degree of confidence in their capacity to carry out the same activities on a different scale, which ranged from 0 (not at all confident) to 10 (extremely confident). Both scales were used to assess identical tasks.

We obtained information on all 109 students from the educational institution they attended so that we could analyze the relationship between stress and academic performance. In particular, we collected information on their grades and the number of credits they received throughout their first two semesters of college, in addition to their enrolment condition for the third semester. The two result variables that the investigators were most absorbed in stood the cumulative grade point average for the first two semesters and the total number of credits earned during that time. A maximum score of 4.0 was used to determine the cumulative grade point average, and students are only allowed to enrol in a maximum of five classes every semester.

### 1.8 Procedures

The coordinator of the freshman seminar obtained permission to distribute questionnaires. The other seminar teachers (with the exception of one) and the coordinator of 11 out of the 12 sections of the course that were taught during that semester said that the surveys' data collection took place in the week before the last one of the spring semesters. Before having the students fill out the surveys, the investigator (who was also one of the authors) went through the informed consent form with them and gave them an outline of what the goals of the research were. After this, the pupils were given an exam that consisted of two pages and took anywhere between 12 and 15 min to complete.

Only nine students out of a total of 129 who were present in the 11 classes where the survey was given decided not to take part in the research. This resulted in a high participation percentage among the responders, which came in at 93.3% overall. On the other hand, we were unable to access the data of thirteen students because the social security numbers or college ID numbers they supplied on the questionnaire were either illegible or absent entirely. In addition, there were five students who did not submit any information on their demographics. Because of this, ten of the participants were taken out of the statistical analysis, leaving a total of 109 students as participants in the sample (see Table 1).

### 1.9 Sample description

The most important features of the sample population are outlined in Table 2, which offers an overview of the data. It is important to note that the age range of the students is highly varied, with 20.7 years being the average age of the first-year students. Based on the average age, it seems that the students participating in this research are about three years older than the first-year students who are typically enrolled in classes.

**Table 1:** Comparison of respondents with Population Features^a^.

	(*n* = 109)	Population
Age	20.7 (3.8)	21.3 (4.5)
Gender		
1. Male	27.1%	34.6%
2. Female	72.9%	65.4%
Race		
3. Chinese	30.8%	27.3%
4. Black	17.8%	17.0%
5. Asian	15.9%	19.7%
Other	–	16.6%

***p < 0.01.

a Population denotes to all undergraduates who registered as ﬁrst-semester freshmen.

**Table 2:** Respondent's features.

Item	Means and Frequencies
**Variables**	
1. Age	20.7 (3.8)*
**Sex**	
2. Male	27.1
3. Female	72.9
Race	
4. White	30.8
5. Black	17.8
6. Chinese	35.5
7. Asian	15.9
8. High school GPA**	3.2 (0.4)
Immigrant status	
9. Chinese	41.1
10. immigrant^a^	36.5
11. Experienced immigrant	22.4
1. College GPA**	2.6 (1.0)
2. Number of credit hours	19.4 (8.8)
(N = 109)	

a been in the Fujian for four years or less.

In terms of the distribution of the sexes among the participants, the sample has a preponderance of females, with around 72.9 percent of the total coming from that demographic. This suggests that there were a greater number of female students participating in the research.

Regarding the racial and ethnic make-up of the sample, Chinese students make up the biggest proportion, accounting for around 41 percent of the total participants. This makes them the most prevalent group. Individuals who self-identified as white made up 30.8% of the total sample, coming in close behind those who identified as Asian. In addition to these groupings, the sample also has a representation of both black and Asian pupils; however, the presented information does not provide particular percentages for any of these groups.

In general, the sample is made up of students who come from a variety of different places, which reflects the varied student population in terms of age, gender, and the racial and ethnic diversity of the student body.

The bottom half of Table 2, which is shown above, provides a summary of the main outcome factors that were discovered during the research. The students' grade point average (GPA) during the first two semesters of college was 2.6 on average. Their highest grade was this one. During this time, the students earned a total of 19 credits on average. It is significant to note that only 35% of the students were able to complete the required 24 credits over the course of two semesters to qualify as full-time students. The remaining students have either chosen to attend school on a part-time basis or have chosen to withdraw from their studies before finishing their first year. In addition to that, during the third semester, thirty percent of the original sample did not show up for any of their courses.

According to the results of simple bivariate correlations, older students have a tendency to have somewhat better GPAs when compared to younger students. This is the case regardless of any other factors. On the other hand, neither the grade point average nor the percentage of students enrolled in the third semester change significantly between male and female students. Comparing students of different ethnicities based on their GPA, we find that Latino students have the lowest average GPA, while Chinese students have the highest average grades in college (2.38 vs. 2.98). The largest dropout rate is seen among students of African descent, who account for 42 percent of all students who withdraw from school by the third trimester.

There is a link between high school grades and college grades, which suggests that students who did well in high school are likely to get better GPAs in college. This is because there is a positive correlation between the two sets of grades. On the other hand, there is an inverse relationship between high school grades and perseverance, which is defined as continuing to register for classes. Students who maintain a higher-grade point average and who acquire more credits have a favorable relationship with one another.

The correlation between grades and the proportion of students who drop out of college is interesting to observe. The grade point averages of students who maintained their education into their second year were, on average, 0.4 points higher than those of students who had quit school the previous year.

The average self-efficacy score was 6.5 out of a possible 27 points with a standard deviation of 1.7 on the tests that measure self-efficacy and stress. The test was taken by 109 pupils. The average stress level, in contrast, is 4.6. Table 3, which presents this data in a table format, ranks the activities that cause the students the most and the least stress. The average level of stress endured by all 109 students was calculated to arrive at these rankings. Writing term papers, having an excessive number of examinations in a single week, and doing well in difficult classes are the three activities that create the greatest stress for students. On the other hand, duties such as establishing friends at school, having conversations with college officials, and comprehending conventions and laws are seen as being less taxing (see Table 4).

**Table 3:** Stressful Responsibilities and their rank scales.

Task	Stress rank	Self-Eﬃcacy rank^a^
Research writing	1 (stressful)	3
Assessments	3	2
Success	2	1 (not conﬁdent)
Building networks	26	16
Interface with faculties	27	19
Indulgent college guidelines	28 (Not stressful)	27 (second most conﬁdent)

a The rank of self-eﬃcacy is in opposite order to enable comparisons.

**Table 4:**
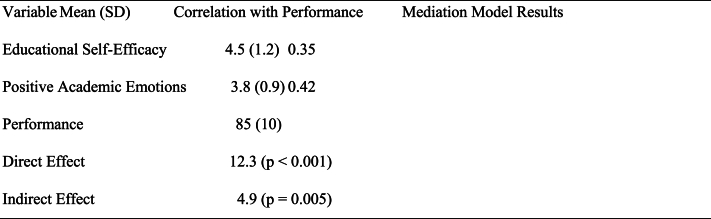
Mediation Analysis educational self-efficacy and performance.

Notably, it is significant to note that the three activities that students selected as the most stressful are also the ones in which they feel the least confidence in their abilities to correctly accomplish. This is an important observation.

It is evident that stress and self-efficacy are negatively correlated, both at the level of the assignment and at the level of the individual student. “To determine whether there was a correlation between the students' stress levels and their results on each of the 28 tasks, we looked at the correlation coefficients.” These statistically significant correlations, which ranged from 0.26 to 0.74, show that there is a close relationship between the two variables.

When they reported feeling less stressed while carrying out certain tasks, they tended to have higher levels of confidence in their ability to successfully execute such tasks. “On the other hand, students who reported greater levels of stress reported considerably lower levels of confidence in their capacity to successfully complete the tasks [7]”.

This research sheds insight on the significant dynamic that exists between stress and perceived levels of self-efficacy. Students will have a greater sense of self-efficacy when they experience less stress in regard to a certain activity. This will lead to an increase in their belief that they are able to successfully complete the work. On the other hand, increased stress levels may diminish students' levels of self-efficacy and impair their confidence in their capacity to do well in academic settings.

The wide variety of correlation coefficients implies that the intensity of the association between stress and self-efficacy may vary depending on the particular job that is being performed. It's possible that certain activities have a somewhat positive link with self-efficacy, which would imply that when stress levels drop, self-efficacy rises to a modest degree. On the other hand, some activities could have a higher positive link, which would indicate a more significant connection between lowered stress and raised levels of self-efficacy.

From the above table it shows that The "Direct Effect" of Educational Self-Efficacy on Performance is estimated at 12.3 (with p-value <0.001), indicating a statistically significant direct relationship. The "Indirect Effect" of Educational Self-Efficacy on Performance through Positive Academic Emotions is estimated at 4.9 (with p-value = 0.005), suggesting that Positive Academic Emotions mediate the relationship between Educational Self-Efficacy and Performance.

In general, these results highlight how important it is to discover effective ways to manage stress in academic contexts. Educators and educational institutions may assist students in developing better levels of self-efficacy by lowering the levels of stress that are associated with certain activities. This, in turn, will contribute to the students' increased levels of self-assurance, motivation, and achievement in their academic endeavors.

## 2 Methods

There were two processes involved in the analysis of the data. To begin, both exploratory and confirmatory factor analyses were carried out in the first stage in order to fulfil the two primary goals that were set. The first thing that we wanted to do was investigate whether or not the questions on the questionnaire that measured stress and self-efficacy could be streamlined into a smaller subset of indices that represent the many facets of each construct. The second thing that we did was look at whether or not stress and self-efficacy could be deemed to be two separate variables based on the methodology that was used to assess them in the survey.

“In the second stage of the study, structural equation modelling was used to evaluate the influence of stress and self-efficacy as latent constructs on the three outcome variables [9]” This was done in order to carry out the second stage of the research. In particular, we focused on the students' grade point averages (GPAs) at the beginning of the third semester after their first year in college. Because stress and self-efficacy are both latent concepts, which means they cannot be directly quantified by the questionnaire questions or the indices that are generated from them, structural equation modelling was determined to be the most appropriate method for conducting this research. In addition, structural equation modelling enables the estimation of cross-equation error correlations, which is a significant advantage. This is significant for the reason that variables like enrollment, credits obtained, and grade point average are anticipated to be connected despite the effects of stress, self-efficacy, or background factors. In addition, it is very important to take into consideration the interconnections between stress and self-efficacy, which were examined by the questionnaire. If you don't estimate different models for each outcome, you might end up with bias as a consequence of an unaccounted-for variable if you don't take into account the correlation of error across equations.

Although structural equation modelling was an effective method for these analyses, extreme care should be taken when interpreting the findings owing to the small number of participants in the study. “This is an essential point to keep in mind. As was said, bigger sample sizes of 100 or, more preferably, 200 or more are often necessary to assure the correctness of the chi-square statistic, as noted in the literature [31].” This is true even when the sample size is greater than the recommended minimum.

In general, the purpose of the two-stage analysis was to first study the component structure of the questionnaire questions related to stress and self-efficacy and then evaluate the impact that these constructs had on the students' GPAs while they were attending college. In spite of the fact that the sample size was restricted, structural equation modelling was able to provide a reliable framework for analyzing these connections; nevertheless, it did recognize the need to exercise care.

## 3 Results and discussions

### 3.1 Factor analysis

It is crucial to remember that exploratory and confirmatory factor analyses reside on a continuum rather than being two distinct methodologies when analysing attitude-related data. It's important to remember this concept. Confirmatory factor analysis tests a predefined factor structure against the data to confirm its fit, whereas exploratory factor analysis allows the data to cluster into factors while considering parameters like rotation method and the number of allowed factors [31]. Data can be clustered into factors using exploratory factor analysis while taking into account variables like the rotation method and the number of allowed factors.

We used a variety of approaches to investigate the factors that relate to stress and self-efficacy. We initially performed exploratory factor analysis by employing a variety of rotation strategies and limiting the overall number of components. We were able to locate potential factor structures contained within the 27 stress elements as a result. Early exploratory studies revealed that the stress items were divided into four separate domains.

From Table 5, its shows that the domains are trouble with school interactions; problems with academic performance outside of class; difficulty with academic performance inside of class; and difficulty with balancing work, family, and school. However, in light of the inadequate loading that four of the items had on the factors, we decided to exclude them from further examination. These things were gaining an understanding of the lecturers, meeting the expectations of one's parents about grades, effectively taking notes in class, and monetary issues.

**Table 5:** Descriptive analysis.

Variable	Mean	Standard Deviation	Range
*Background Variables*			
1) Age	20	3	17–34
Male	0.26		
2) Non white	0.70		
3) immigrant	0.37		
4) Eng	0.47		
a) GPA	3	0.46	2–4
*Stress Scales*			
5) Collaboration *	3	2	0–9
6) Work routine out of lecture**	5	2	0.12–9.6
7) Class presentation in session	6	2	0–11
8) Balancing work, family, and school	4	2	0.5–9.7
*Self-Eﬃciency Scales*			
9) Collaboration *	6	2	1.54–10
10) Work routine out of lecture**	6	1.90	0.65–11
11) Class presentation in lecture	6	2.19	1–11
12) Balancing work, family, and school	6	1.92	1–10
*Outcome Variables*			
GPA	2	0.97	0–4
Credits	19	8.82	0–36
Enrolled	0.72		
*N* = 109			

We performed confirmatory analyses once the exploratory study was finished to determine how well the variables identified in those studies matched the data. The findings indicated that only a few changes were necessary to these components. We also conducted a second assenting factor analysis to verify the accuracy of the results for the self-efficacy items. “Similar to the stress items, the self-efficacy questions were divided into four different categories: confidence in academic performance inside of the classroom, confidence in academic performance outside of the classroom, confidence in maintaining a healthy balance between work, family, and academic responsibilities [19].” Table 4's findings from the confirmatory factor analysis showed that these four factors were accurate, with the first factor having a loading of seven items, the second factor having a loading of eight items, and the other two factors having a loading of four items each.

Using a combination of exploratory and confirmatory factor analyses, we were able to identify and validate the component structures for both the stress and self-efficacy items. Both of these studies were beneficial to us. Due to their influence on the interactions between these parameters, these studies provide a solid foundation for further investigation of the relationships between these variables and their impact on the outcomes of interest.

Taking into consideration the phrasing of the questionnaire questions and the similarity in factor loadings, we next investigated whether or not the stress factors and their related self-efficacy components displayed a full negative connection. “Because the same questions were used to evaluate both stress and self-efficacy, it is likely that these two concepts reflect opposing ends of a continuum. More specifically, it is possible that people who experience greater levels of stress display lower levels of self-efficacy (possibly in coping with that stress) [8].” In order to investigate the nature of this connection, we carried out confirmatory factor analyses using two components: one component stood for stress, while the other component stood for self-efficacy.

“The findings of these studies provide additional proof that stress and self-efficacy are not simply opposite ideas to be considered in opposition to one another. According to the results of chi-square difference tests, there was a statistically significant disparity in the degree to which the constrained (completely correlated) and unconstrained (partially correlated) models fit the data [10].” This lends credence to the idea that stress and self-efficacy are related factors that, although evaluated in a comparable fashion within the survey, continue to retain their individual qualities.

In conclusion, the results of our research lend credence to the theory that stress and self-efficacy are interrelated but distinct concepts. Although they may seem to be competing forces based on the questionnaire questions and factor loadings, they really exhibit separate relationships and play various roles in people's experiences. This is despite the fact that they may appear to be competing forces.

### 3.2 Structural equation modeling

We pooled the data and produced averages for each element in order to enable structural equation modelling. This led to the creation of four stress indexes and four self-efficacy indexes. In the structural equation models, these indices served as indicators for the general stress component and the general self-efficacy factor. This strategy was selected for a couple of different reasons. To begin, we were unable to estimate a model with eight independent components due to the small size of our sample population. The number of parameters exceeded the amount of information that was readily accessible in the data. Second, the findings that were produced using this method continued to be consistent with other robustness tests, which made it a choice that was simpler when compared to the option of estimating a model that was more complicated.

Cronbach's alphas, which are shown in Table 6, were used in order to conduct an analysis of the indexes' level of consistency. Alpha values across all indices ranged from 0.72 to 0.90, indicating that the level of consistency was adequate.

**Table 6:**
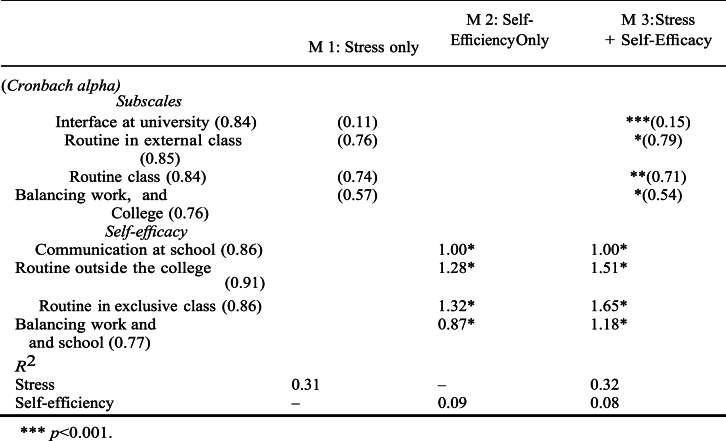
Results of Structural Equation Models.

***p < 0.001.

**Fig. 1:**
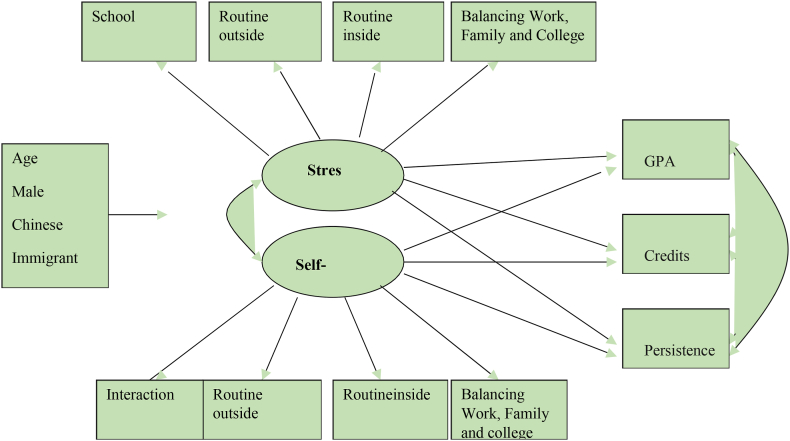
Path Diagram.

Fig. 1 is a visual representation of the total structural equation model, which includes the background and control factors as well as the outcome variables. These background and control variables include things like age, gender, race, nativity status, language spoken at home, and high school GPA. “These variables were included in the model as potential factors that might have an effect on both stress and self-efficacy. Indicators for the latent dimensions of stress and self-efficacy were provided via the composite indices that were discussed before [24].” It was hypothesised that stress and feelings of self-efficacy would be able to predict each of the three outcome factors.

“According to the findings of the measurement model, each of the four indicators of stress and the four indications of self-efficacy had a substantial impact on the latent variables to which they were related [22]” Notably, the first indication, which assessed interaction at school, demonstrated poorer reliability in comparison to the other indicators, indicating a less reliable assessment for both stress and self-efficacy. This finding is noteworthy.

“We used three different structural models to analyze the relationship between academic results and characteristics like stress, self-efficacy, and background factors.” “The first model concentrated on stress and background variables, whereas the second model focused on self-efficacy and background factors, and the third model incorporated both stress and self-efficacy in addition to background components [29].” “All three of the models were able to satisfactorily describe the data when they were evaluated using the chi-square, the root mean squared error of approximation (RMSEA), and the incremental fit index (IFI).”

We found no association between stress and any of the three outcomes using the first model, which took into account both stress and background variables. There were very few demographic characteristics in the background that had a substantial influence. The chance of continuing one's education beyond high school was marginally impacted less favorably by one's grade point average. At the conclusion of the first year, non-Chinese students had a lower number of credits and a worse grade point average. New immigrants and students who had better grade point averages in high school had higher first-year college grade point averages. The amount of variation in persistence, credits, and grade point averages that might be attributed to stress and background factors varied from 9 to 23 percent. Furthermore, there were moderate and substantial error correlations across the outcome measures. This suggests that GPA, persistence, and credit accumulation were significantly connected even after accounting for alternative factors that may have caused confusion.

The self-efficacy and background elements were the only foci of attention in the second model. After the third semester, self-efficacy had little influence on perseverance, but it did have a substantial beneficial effect on both credits earned and grade point average. A small number of background characteristics were shown to be related to any result, much as the previous model found. There was a marginally significant inverse connection between persistence and both the high school grade point average and the usage of Chinese as the primary language spoken at home. Students who were not of Chinese descent earned a lower total number of credits than their Chinese counterparts.

“In the third model, which incorporated background variables, stress, and self-efficacy, self-efficacy had no influence on persistence, whereas stress had a marginally significant impact that was positive [9].” “However, self-efficacy did have an effect on both the number of credits earned and the grade point average. The influence of background characteristics on the outcomes remained very minor, with high school GPA having a favorable effect on credits and college GPA having a tiny negative effect on perseverance [17].”

“The correlation between stress and self-efficacy explained 33% of the variation in grade point average, 15% of the variance in the number of credits earned, and 10% of the variance in future studies [13].”

“The relationship between levels of perceived stress and academic self-efficacy in relation to predicting academic success among first-year students was the focus of this study's main premise [22]”. To gauge self-efficacy and stress related to college activities, a survey instrument was developed. This made it possible to evaluate these elements' effects on nontraditional students' grades, credit accrual, and ability to continue attending college. Both measurements showed acceptable amounts of internal consistency. “Stress and academic self-efficacy both showed a negative relationship, which is consistent with earlier research. Both scales exhibited equivalent components, according to the results of the factor analyses, which is consistent with the self-domain-specificity of the self-efficacy idea [32]”. The article emphasized the relative impact of stress and self-efficacy in predicting academic results and noted that self-efficacy consistently surpassed other indicators in predicting GPA. In contrast to the results of other studies, some of the data given here suggested that stress and persistence might have a very slight positive correlation. The results of this study highlight how important it is to distinguish between identifying threats and dealing with obstacles when trying to understand how stress impacts academic performance.

## 4 Discussion and implications

Our study delved into the connections between educational self-efficacy, academic emotions, and academic performance. The findings demonstrated clear and significant associations: self-efficacy exhibited a positive relationship with both positive academic emotions and academic performance. Notably, mediation analysis unveiled that positive academic emotions act as intermediaries in the link between self-efficacy and academic performance. These results resonate with Bandura's Social Cognitive Theory, reinforcing the importance of nurturing self-efficacy and cultivating positive emotional experiences in educational settings. Practical implications emerge, suggesting that educational institutions and educators can proactively enhance self-efficacy and create an environment that fosters positive academic emotions to ultimately improve academic outcomes.

### 4.1 Future research

This study offers valuable insights but also highlights avenues for further exploration. Future research could take a longitudinal approach to understand how these variables evolve over time. Cultural and contextual variations should be considered to appreciate the diversity of student experiences. Investigating the effectiveness of specific interventions designed to boost self-efficacy and promote positive academic emotions is crucial. Moreover, the study could delve into the nuances of emotional processes that mediate the relationship between self-efficacy and academic performance, potentially uncovering more complex pathways. Furthermore, exploring additional mediators that connect self-efficacy to academic performance can provide a comprehensive view of these dynamics.

## 5 Conclusion

Using our findings, there are some things that cannot be considered. First of all, the study only included a small number of individuals, which may make it challenging to generalise the findings. Since all of the participants were first-year students who volunteered to attend the seminar, there may also be an issue with the sample's capacity to correctly represent the population. If students' academic success and their desire to continue their study were affected by attending the seminar, our results might be biased. “On the other hand, findings from earlier studies imply that participation in the seminar may not have a major influence on students' ability to adapt to the rigours of college [32].”

Despite these limitations, the discoveries we have uncovered open up crucial new viewpoints. “We discovered that while academic self-efficacy had a considerably greater effect on forecasting the accumulation of college credits and a higher-grade point average than perceived stress, which may have a small impact on predicting future enrolment. This observation is in line with patterns that are frequently discussed in the literature referenced above [33,34].” “The ability of academic self-efficacy to lessen the impact of stressors on students' perceived levels of stress is also highlighted as being important in predicting academic success in college. It also emphasises how important academic self-efficacy is in predicting academic success in college [6].”

“Our study focuses on the importance of academic self-efficacy as well as the general significance of students' perceptions of their ability to thrive academically.” This demonstrates that fostering a sense of self-assurance in children may have a positive effect on their academic progress. Additionally, since perceived stress has so little of an impact on academic achievement, stress management and coping mechanisms must be addressed in order to improve students' overall wellbeing and academic success.

It is essential to emphasise that the population of freshmen students that took part in the lecture was particular to the conclusions that we obtained by analysing their participation. To confirm and generalise the findings of this study, we would need more research with populations that are both larger and more varied. In spite of this, our research makes a contribution to the current body of knowledge by illuminating the relative significance of perceived stress and academic self-efficacy in predicting academic achievement, as well as by giving insights into possible treatments to improve students' success in college.

## 6 Limitations

Several limitations warrant consideration. The sample size of 109 participants, while reasonable, may not fully represent the diversity within the broader student population. The reliance on self-report measures for self-efficacy and emotional experiences might introduce response biases, and the inclusion of objective measures could enhance the quality of the data. Additionally, the cross-sectional nature of the study limits our ability to establish causality. To address this, future research could employ experimental designs or longitudinal approaches. The generalizability of the study may be constrained by the specific educational context and the characteristics of the sample, emphasizing the importance of replicating the study in diverse settings. Lastly, the mediation model presented in this study is simplified; real-world processes likely involve multiple mediators and moderators, necessitating further exploration of these complexities.

In summary, our study provides valuable insights into the intricate relationships between self-efficacy, positive academic emotions, and academic performance. It underscores the need for further research to address these limitations and explore the multifaceted nature of these relationships.

## Funding

This study was supported by Humanities and Social Sciences Project of the Ministry of Education (Grant ID:19YJC740046)

## Data availability statement

Data will be made available on request.

## CRediT authorship contribution statement

**Wenwen Cao:** Writing – review & editing, Writing – original draft, Supervision, Funding acquisition, Conceptualization. **Sanga Mithra S. Gnana:** Writing – review & editing, Writing – original draft, Validation, Methodology, Investigation, Data curation. **Aravind B R:** Writing – review & editing, Supervision, Resources, Methodology, Formal analysis.

## Declaration of competing interest

The authors declare that they have no known competing financial interests or personal relationships that could have appeared to influence the work reported in this paper.
