# Supplementary material for: Unraveling the factors shaping academic success: A structural equation modeling approach for college students
Source: Heliyon. 2024 Feb 9;10(4):e25775. doi: 10.1016/j.heliyon.2024.e25775 (PMC10875422; doi:10.1016/j.heliyon.2024.e25775)
Supplement: Multimedia component 1 [file mmc1.docx]

**Measurement items in a Questionnaire**

| **Constructs** | **Variables** |
| --- | --- |
| **Fear of delay** | 1. I’ am afraid I can’t graduate in 3 years 2. I ‘am afraid that I won’t be able to finish my degree |
| **Self- efficiency** | 1. I’ am very focused on my education 2. I think I did my best 3. I have no plans to drop out 4. Over all I am enjoying education |
| **Parent’s Support** | 1. My parents have always supported me emotionally 2. My parents encouraged me to achieve 3. Parents supported me to have work study balance |
| **Teachers Support** | 1. Our lecturers are fair 2. Our lectures encouraged me 3. They accepted our ideas and plans |
| **Stress level** | 1. Duties and responsibilities make me emotionally depressed. 2. I felt exhausted throughout 3. Education makes me stressed 4. I lost enthusiasm for learning 5. I have thought of dropping out |
| **Academic Performance** | 1. I got satisfactory grades 2. I got a lot of knowledge for my future while interacting with teachers 3. My ability to study increased during my course. 4. I feel that my critical thinking skills have increased while pursuing my course. |
